# LDL-Cholesterol Lowering of Plant Sterols and Stanols—Which Factors Influence Their Efficacy?

**DOI:** 10.3390/nu10091262

**Published:** 2018-09-07

**Authors:** Elke A. Trautwein, Mario A. Vermeer, Harry Hiemstra, Rouyanne T. Ras

**Affiliations:** Unilever R & D Vlaardingen, Olivier van Noortlaan 120, 3133 AT Vlaardingen, The Netherlands; mario.vermeer@unilever.com (M.A.V.); harhiem@xs4all.nl (H.H.); rouyanneras@hotmail.com (R.T.R.)

**Keywords:** plant sterols, plant stanols, phytosterols, LDL-cholesterol lowering, food format, intake occasion, intake frequency, meal composition, gender, age

## Abstract

The LDL-cholesterol (LDL-C) lowering effect of plant sterols/stanols (PSS) is summarized in several meta-analyses showing a dose-response relationship with intakes of 1.5 to 3 g/day lowering LDL-C by 7.5% to 12%. This review summarizes evidence for the impact of various factors potentially influencing the LDL-C-lowering efficacy of PSS. PSS are efficacious in all food formats and in food supplements. Some factors related to food format, e.g., solid vs. liquid foods, seem to impact efficacy, while there is no difference between free PSS and esters. Compared to multiple daily intakes, once-a-day intake of PSS, especially in the morning with light breakfast, leads to a sub-optimal LDL-C lowering. However, intake frequency seems influenced by intake occasion, i.e., with or without a meal, and time of day. Meal intake is a critical factor for an optimal LDL-C lowering efficacy of PSS. While age has no impact, gender is suggested to influence the LDL-C lowering effect of PSS with greater reductions reported for men than women; but overall evidence is inconclusive and larger studies show no gender by treatment interaction. In conclusion, PSS are efficacious in all foods and food supplements; for optimal efficacy they should be consumed with a (main) meal and twice daily.

## 1. Introduction

Plant sterols (PSter) and stanols (PStan), also known as phytosterols (here collectively being abbreviated as PSS), are cholesterol-like compounds that occur naturally in plant-based foods. Food sources of PSS are vegetable oils, vegetable oil-based margarines, seeds, nuts, grain products, vegetables, legumes and fruits next to various food formats and food supplements with added PSter or PStan. PSS intake from natural sources ranges between 200 and 400 mg/day with habitual diets [1] and up to 600 mg with vegan- or vegetarian-type diets [2]. Higher intakes can only be achieved by consuming typical servings of food products enriched with PSS such as fat-based spreads and margarines or dairy-type foods like milk, yogurt and yogurt drinks. PSS contents of such foods are typically 0.75 to 2 g per serving size.

The LDL-cholesterol (LDL-C) lowering effect of PSS has been summarized in several meta-analyses based on a vast number of randomized, placebo-controlled clinical studies. The Katan et al. meta-analysis [3] forms the first landmark paper substantiating the LDL-C lowering effect of PSS. Further meta-analyses were published in subsequent years addressing a continuous dose-response relationship and the impact of the food format on the cholesterol lowering efficacy of PSS [4,5,6]. The most recent meta-analysis by Ras et al. [7] included 124 clinical studies (with 201 strata) and over 9600 study participants. The average PSS intake was 2.1 g/day (range 0.2–9.0 g/day), and overall, a consistent dose-response relationship for lowering LDL-C by 6–12% with intakes of 0.6–3.3 g/day was found. 

A tapering-off effect for the LDL-C-lowering effect of PSS is expected as the inhibition of cholesterol absorption is a saturable process [8,9], but probably only at intakes higher than 3 g/day. However, as the number of clinical studies with intakes greater than 4 g/day is rather limited, it remains speculative whether the dose-response relationship would continue and, further, whether efficacy would differ between PSter and PStan at higher doses as was previously suggested [6]. At intakes up to 3.3 g/day, PSter and PStan similarly lower LDL-C concentrations as established by meta-analyses including one that included only clinical studies that compared PSter and PStan side by side [7,10].

An overview of the relative LDL-C lowering of PSS as summarized in meta-analyses is presented in Table 1 showing the dose-response relationship of increasing intakes of PSS lowering LDL-C by up to 12%.

Many factors related to the food format may influence the cholesterol-lowering efficacy of PSS. Such factors include a liquid or solid food matrix, the fat content and fat type of the food, a supplement, i.e., capsule or tablet, versus food format, the use of free versus esterified PSS or the fatty acids used for esterification. Other important factors are frequency of administration, e.g., single vs. multiple daily intakes, intake occasion related to intake with or without a meal and time of administration during the day, e.g., morning vs. later during the day.

Next to food format or matrix effects and intake-related factors, other more metabolically-related influences are thought to affect the cholesterol-lowering efficacy of PSS such as age, gender, health vs. disease status and being classified as ‘cholesterol absorber’ or ‘cholesterol synthesizer’ based on cholesterol absorption or synthesis efficiency. Regarding the latter, it has been shown that a phenotype characterized by high cholesterol absorption and low cholesterol synthesis affects the responsiveness to PSS intake and that genotypic variations in genes of cholesterol metabolism may play a role [8,9,10]. Responsiveness, i.e., being a responder or non-responder to PSS intake has been addressed in detail in previous publications [8,9,10].

The LDL-C lowering effect of PSS in different populations including both healthy and diseased populations such as with familial hypercholesterolemia (FH), type-2 diabetes mellitus (T2DM) or Metabolic Syndrome (MetSyn) has been studied in many intervention studies as summarized by Plat et al. [11]. It was concluded that PSS are effective in both healthy and diseased individuals. However, the available evidence for an equal efficacy in diseased compared to healthy populations is rather limited because of lesser intervention studies carried out in populations with FH, T2DM and MetSyn. A recent study has further strengthened the evidence that PSter lower plasma lipids in individuals at risk of and with established T2DM who have elevated basal triglyceride (TG) and LDL-C concentrations. An intake of 2 g/day of PSter significantly lowered both LDL-C and triglyceride (TG) concentrations even when taking statins [12].

In the following parts of this review, the impact of food format, intake occasion and frequency as well as of age and gender on the cholesterol-lowering efficacy of PSS will be discussed.

## 2. Impact of Food Format/Matrix

**Figure 1:**
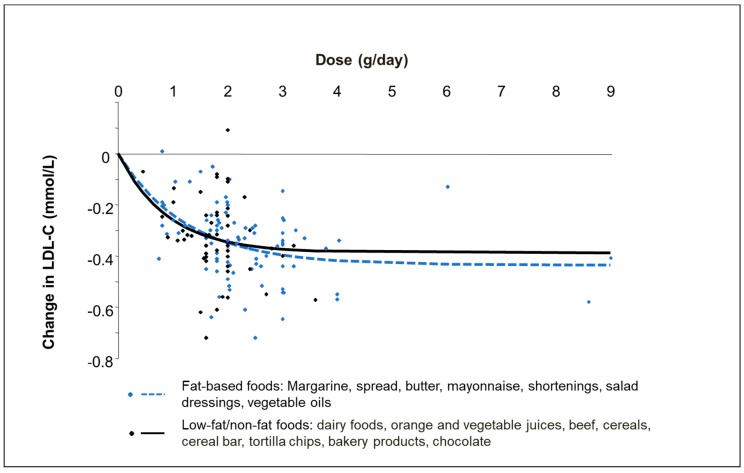
Dose-response curves of the absolute LDL-cholesterol (LDL-C) lowering effect of (combined) plant sterols and stanols added to fat-based foods as compared to low-fat/non-fat foods (based on meta-analysis of Demonty et al. 2009 [5]).

Fat-based products like margarine and spreads next to dairy-type foods such as milk, yoghurt, and yoghurt drinks are the most common food formats used for enrichment with PSS. Whilst most clinical studies have been carried out with these food formats, many studies have also investigated the cholesterol-lowering effect of other food formats including mayonnaise, salad dressing, soy products, cereals, bakery products, orange juice and vegetable oils [4,5]. Based on evidence from two meta-analyses, PSS are effective in all food formats with no apparent difference in the LDL-C lowering efficacy between fat-based and low- or non-fat based foods [4,5]. AbuMweis et al. [4] reported that PSS added to fat-based spreads, mayonnaise and salad dressings had a similar LDL-C lowering effect as dairy foods with an effect size of −0.32 to −0.34 mmol/L (95% confidence interval (CI): −0.25, −0.40 mmol/L). This was thereafter confirmed by Demonty et al. [5] showing that the established dose response curves for fat-based foods compared to low-fat or non-fat foods did not differ (Figure 1). Nevertheless, a tendency for a higher efficacy of solid/edible foods, i.e., predominantly fat-based products like spreads and margarines vs. liquid/drinkable food formats such as milk and juices, was found in two meta-analyses [5,7]. Demonty et al. [5] found that at doses >2 g/day, the maximal LDL-C lowering effect was 5.2% larger for solid than for liquid foods; however, the difference in LDL-C lowering at typical PSS intakes of 1.5–2 g/day was small. Ras et al. reported that in the dose category of 2.0 to 2.5 g/day (*n* = 60 studies), liquid food formats (*n* = 21 studies) lowered LDL-C concentrations by on average 6.5% compared to 9.2% (*p* = 0.003) for solid foods (*n* = 39 studies) [7]. An explanation why liquid food formats might be less efficacious than solid foods may be related to faster gastric emptying and hence faster transit time in the gastrointestinal tract where the main mechanism of action of PSS takes place. Likewise, liquid foods may not always be consumed together with a meal or as part of a meal. Sufficient ingestion of food, i.e., of fat, is required to trigger bile release for PSS to optimally compete with cholesterol for micellar incorporation and subsequently to optimally inhibit cholesterol absorption [13]. Still, there are too few head-to-head studies comparing fat-containing vs. low-fat/non-fat foods or comparing solid vs. liquid foods in the same clinical study setting [14,15] to draw final conclusions on possible food matrix effects.

Whether the type of carrier fat used in the formulation of PSS-added foods could impact the LDL-C lowering effect was studied in a recent meta-analysis [16]. Three types of carrier fat, namely rapeseed/canola oil, soybean/sunflower oil or dairy fat were included in this meta-analysis of 32 studies including 2157 participants. The PSS intake ranged from 1.5 to 4.0 g/day and the food format comprised mainly spreads and margarines and dairy-type foods like milk, yogurt, butter and cheese. No difference in the relative reduction in LDL-C between the different fat carrier groups was found (range: 8.9 to 9.7%); the overall 9% reduction is well in line with effects found in other meta-analyses [7]. Nevertheless, a significantly larger absolute reduction in LDL-C concentrations was found with the rapeseed/canola oil formulations (−0.38 mmol/L, 95% CI: −0.43, −0.34) as compared to the soybean/sunflower oil formulations (−0.28 mmol/L, 95% CI: −0.35, −0.22). Why rapeseed/canola oil as a carrier fat would be superior over sunflower/soybean oil remains unclear. In fact, it could be assumed that a polyunsaturated fatty acid (PUFA)-rich soybean/sunflower oil would give a larger LDL-C lowering compared to a monounsaturated fatty acid (MUFA)-rich rapeseed/canola oil. Based on meta-analysis evidence, replacing 5% energy of saturated fats with PUFA leads to a larger reduction in LDL-C than replacement with MUFA [17].

Whether MUFA or PUFA might influence the incorporation of PSS into micelles as suggested by the authors seems hypothetical [16]. Notably, the results of this meta-analysis seem confounded by differences in PSS intakes between the fat carrier groups as the average intake was with 2.6 g/day higher in the rapeseed/canola oil group compared to 2.1 to 2.2 g/day in the two other fat carrier groups. Possibly, publication bias (lack of studies) could be another confounder since only studies that reported details of the fat carrier used in the food formulations were included in this meta-analysis. 

### 2.1. Foods Versus Food Supplements

The LDL-C lowering effect of capsules or tablets has so far been studied in a limited number of clinical studies. For instance, the meta-analysis of Ras et al. includes just six studies with a supplement format [7]. A meta-analysis involving exclusively studies with PSS in supplement form, included just eight studies (nine strata) administering different types of PSS formulations. It was concluded that there was no significant difference in the LDL-C lowering effect of PSS supplements (−0.31 mmol/L, 95% CI: −0.39; −0.23 mmol/L) compared to foods (−0.31 mmol/L, 95% CI −0.35; −0.27 mmol/L) [18] at similar intakes. This reported mean LDL-C lowering effect is furthermore comparable to those reported in other meta-analyses [5,7]. 

Considering supplements, it should however be noted that tablets and capsules often contain different formulations of PSter or PStan either in their free or esterified form as well as lecithin complexes. These formulations may differ in their particle size, often not reported in publications, and this can have an impact on the cholesterol-lowering efficacy. It is generally believed that large-sized plant sterol crystals may not be efficient at reducing the absorption of cholesterol in vivo, whilst non-crystalline preparations and preparations containing micro-sized crystals will be efficient [19]. 

Further, it remains important to also look at the disintegration time of supplements. In a head-to-head comparison study, slowly disintegrating capsules (60 min) and rapidly disintegrating tablets (5 min) were compared, both containing a spray-dried PStan lecithin complex [20]. Administering 1.3 g/day PStan with the rapidly disintegrating tablets lowered LDL-C by 10.4% as compared to a non-significant 2.5% with the slowly disintegrating capsules delivering 1.0 g/day PStan [20]. 

Overall, the LDL-C lowering effect of supplements is comparable to that of foods with added PSS, there are also a few clinical studies that failed to lower LDL-C using capsules with PStan or PSter either in free or esterified form [21,22]. Reasons for this lack of LDL-C lowering are not well understood but could be related to the type of formulation used, as explained above. Clearly, it is critical to better understand the impact of the dose-delivery system because an optimal disintegration time of the supplement, particle size of PSS formulations as well as possible crystal formation and growth may be important factors that influence supplement efficacy. Head-to-head comparison studies would be needed to fully conclude on equality or even the superiority of specific supplement formulations or a potential difference in efficacy between a food matrix and a supplement. Furthermore, still many published studies provide no information about particle size or disintegration time of the PSS formulations used in capsules or tablets; such information would be prudent to have. 

## 3. Impact of Plant Sterol and Stanol Source

The impact of PSS source on the LDL-C-lowering effect of PSS has not been exclusively investigated in meta-analyses. Only a few individual studies have considered this aspect as summarized below. Clifton et al. [23] compared the LDL-C lowering effect of esterified PSter from either soybean oil, tall oil or a mix of tall and rapeseed oil. Three weeks intake of 1.6 g/day followed by 3 weeks of 3 g/day esterified PSter led to comparable reductions in LDL-C of 7–11% between the different PSter sources. It was therefore concluded that different sources of PSter are equally effective [23]. A clinical study investigating the effects of spreads containing 2 g/day PSter from either rapeseed oil or tall oil, found comparable LDL-C lowering effects of 8–9% [24], while only rapeseed oil PSter seemed to also beneficially affect markers of endothelial function and hemostasis (i.e., E-selectin and total plasminogen activator inhibitor-I (tPAI)) [25]. Hence, limited evidence suggests that differences in the PSter composition due to use of different sources have no impact on the cholesterol-lowering efficacy. Whether different sources affect endothelial function and hemostasis markers differently requires further investigation.

### 3.1. Free Versus Esterified Plant Sterols/Stanols

Esterification of PSS with dietary fatty acids may facilitate their incorporation into micelles, and hence, it seems plausible that esterification could be important for the cholesterol-lowering efficacy of PSS [26]. Clinical studies comparing head-to-head un-esterified (free) vs. esterified PStan added to spreads, breakfast cereals and bread [27] or water-dispersible free PSter vs. PSter esters [28] have shown comparable LDL-C lowering effects. Another study comparing different forms (1.7 g once a day with breakfast consumed under supervision) including free PSter and PSter esterified to fatty acids from sunflower oil or fish oil added to margarine in the same study setting, failed to show any cholesterol-lowering effect compared to control treatment [29] and could thus not be used to assess potential differences between free and esterified PSter. Based on this finding, it was hypothesized that time of intake, e.g., in the morning, may lead to suboptimal efficacy as discussed in detail below. 

A comparable effect of free PSS vs. PSS esters has also been confirmed by two meta-analyses [5,7]. The most recent meta-analysis by Ras et al. [7] compared 45 studies with free PSS to 152 studies with PSS esters. LDL-C concentrations were reduced by 7.5% (95% CI: −8.7; −6.3) with free PSS and by 8.7% (95% CI: −8.7; −6.3) with esterified PSS (*p* = 0.063). This non-significant difference could be related to the difference in the daily intakes with 1.5 g/day for free vs. 2.2 g/day for esterified PSS. Of note, PSS esters are most often added to fat-based products like spreads and margarine because of their higher fat solubility, while free PSS are usually used in a variety of low-fat or non-fat foods including liquid foods.

### 3.2. Impact of Fatty Acids Used for Esterification

Most commonly, PSS are esterified with either oleic or linoleic acid from common vegetable oils like rapeseed (canola), sunflower or soy bean oil. A limited number of clinical studies has assessed the impact of other fatty acids used for esterification of PSS on their cholesterol-lowering efficacy. For instance, using stearic acid for esterification appeared to lead to a comparable cholesterol-lowering effect as with common PSS esters, although the clinical study by Carr et al. did not include PSS esterified with oleic or linoleic acids for direct comparison [30]. Furthermore, head-to-head comparison studies comparing fish oil, sunflower and olive oil PSter esters also found comparable LDL-C lowering effects [31,32]. Thus, the choice of fatty acids used for esterification does not seem to have an impact on the cholesterol-lowering efficacy of PSS. 

Nevertheless, the rate of hydrolysis of the PSS esters ranges from 40 to 96% (average 73% for PStan esters and 80% for PSter esters) amongst individuals and seems to be affected by the fatty acid moiety of the ester and lesser by the PSter or PStan moiety [33]. For oleic, linoleic and α-linolenic acid, comparable hydrolysis rates were reported, whereas palmitic, stearic, eicosapentaenoic acid or ferulic acid from oryzanol found in rice bran were hydrolyzed at a lower rate due to substrate specificity of cholesterol esterase [33,34]. Hydrolysis of PSS esters into free PSS in the intestinal lumen is an important first step in the mechanism of action of cholesterol absorption inhibition.

Evidence for novel PSS formulations, such as alternative esters like a disodium ascorbyl phytostanyl phosphate analogue [35] or a triglyceride-recrystallized PSter complex [36,37] is still too scarce to conclude on a comparable efficacy of established formulations with free and esterified PSter and PStan. 

## 4. Impact of Intake Frequency and Intake Occasion (without/with Meal and Time of Day)

So far, only one clinical study has explicitly assessed the impact of intake frequency by comparing once-a-day vs. three-times a day intake [38]. It was concluded that there was no difference in the LDL-C lowering effect after consuming 2.5 g/day of PStan either once with lunch or three times with breakfast (0.42 g), lunch (0.84 g) and dinner (1.25 g). Hence, consuming PSS only once-a-day or several times daily seems not to affect efficacy. Nevertheless, data from two meta-analyses came to a different conclusion when comparing the LDL-C lowering effect of studies administering PSS once-a-day vs. two or more times a day (Table 2) [5,7]. In these meta-analyses, higher relative reductions in LDL-C were found with multiple daily intakes of PSS as compared to once-a-day intakes. Although intake frequency seems to influence the cholesterol-lowering efficacy of PSS with once-a-day intake being sub-optimal, it should be noted that these findings may partly be influenced by intake occasion, i.e., intake with or without a meal and time of day (see below).

The meta-analysis of AbuMweis et al. [4] assessed the impact of intake frequency defined as once vs. 2–3 times a day whilst, at the same time, considering the intake occasion. Interestingly, PSS intake once-a-day in the morning (and probably without a sufficient meal) was found to result in a suboptimal LDL-C lowering effect. While comparable LDL-C lowering effects were found for intakes of 2–3 time/day (−0.34 mmol/L, 95% CI −0.38; −0.30 mmol/L) and once-a-day intake in the afternoon or with a main meal (−0.30 mmol/L, 95% CI −0.39; −0.21 mmol/L), a once-a-day intake in the morning resulted in a lower efficacy of −0.14 mmol/L (95% CI −0.29; 0.00 mmol/L).

Based on post-hoc analyses of the data from the most recent meta-analysis [7], Ras et al. assessed the impact of intake frequency and intake occasion similarly as done by AbuMweis et al. [4] (Table 3). Especially, once-a-day intake of PSS with breakfast seems to lead to a lesser LDL-C lowering effect of PSS as compared to a daily intake of more than once-a-day. However, differences in PSS intakes ranging from 1.7 to 2.2 g/day between the groups may have partly confounded this finding. 

Noteworthy, many clinical studies still do not report information on intake frequency and intake occasion; more details on how PSS were administered in clinical studies would be useful for further clarifying the impact of intake occasion and frequency in future meta-analyses. Nevertheless, so far intake occasion and frequency seem to be critical aspects for an optimal LDL-C efficacy of PSS. 

Is there an explanation why once-a-day (in the morning) would lead to a sub-optimal cholesterol lowering effect? 

Inhibition of the intestinal absorption of exogenous (dietary) and endogenous (biliary) cholesterol is the main underlying mechanism for the cholesterol-lowering effect of PSS. 

Several underlying mechanisms contribute to the overall inhibition of intestinal cholesterol absorption by PSS [39,40]. A key mode of action is considered the displacement of cholesterol by PSS from the mixed micelles due to their limited capacity to embody sterols next to their interference with transporter-mediated processes of cholesterol uptake. 

It was previously hypothesized that a reason for a lesser efficacy of a once-a-day PSS intake especially in the morning may be related to a circadian rhythm of cholesterol absorption [4]. A circadian rhythm is well established for cholesterol synthesis with maximal synthesis around midnight and minimal synthesis around noon time [41,42]. Also, for bile acid synthesis such a circadian rhythm has been described with maximal synthesis during daytime and minimal during night [43]. However, no clear evidence for a circadian rhythm of cholesterol absorption has so far been reported [44]. It seems therefore unlikely that a circadian rhythm of cholesterol absorption may play a role in the lower efficacy of a single PSS intake consumed with breakfast. 

Stimulation of bile flow as prompted by food intake is a crucial step in the formation of mixed micelles and plays an important role in the overall mechanism of action. Hence, an explanation for a sub-optimal effect of a once-a-day intake during breakfast could relate to insufficient food intake and thus insufficient interference with cholesterol uptake. The importance of consuming PSS with a (main) meal has already been suggested before [4,11], and was further demonstrated in head-to-head comparison studies [45,46]. A larger LDL-C lowering effect of 9.4% was found when a yoghurt drink with added PSter was consumed together with a lunch meal compared to a 6.0% lowering when the yoghurt drink was consumed before breakfast on an empty stomach [45]. A study with PStan-enriched biscuits also found that consumption with a meal resulted in a greater cholesterol-lowering effect compared to consumption between meals [46]. Furthermore, two studies carried out by the same investigators testing a similar yoghurt drink providing the same PStan intake (2 g/day) have shown a LDL-C lowering of 11.8% when the yoghurt drink was consumed with lunch under supervision [47], but a low efficacy (−3.2%) when the yoghurt drink was consumed with lunch but with no supervision, and thus without guaranteed meal intake [47]. Evidently, intake with a meal and possibly also meal composition enhances the cholesterol-lowering efficacy of PSS.

**Figure 2:**
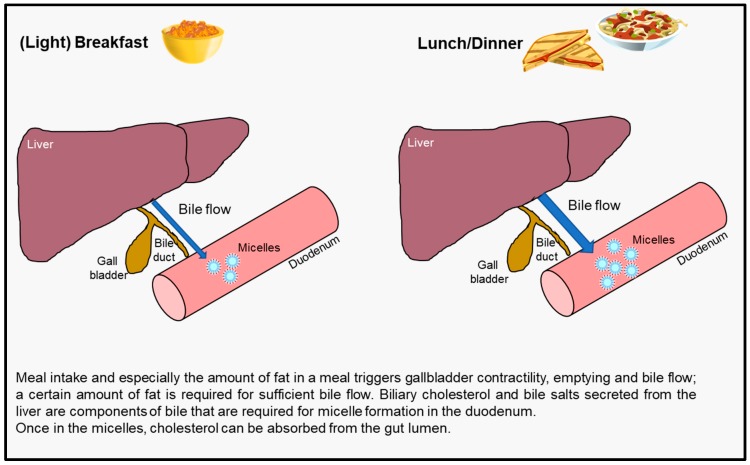
The impact of meal intake on gallbladder contractility and bile flow affecting micelle formation in the gut.

An intubation study collecting duodenal content samples after meal intakes [48] measured bile salt concentration in the duodenum and showed that intake of a yoghurt drink with added PSter did not stimulate duodenal bile flow when consumed without a meal while intake together with a meal clearly stimulated bile flow [13]. No or limited bile flow means there is no or minimal release of biliary cholesterol and bile acids into the gut lumen, both being components of micelle formation. Thus, inhibition of cholesterol absorption may be lessened if a food with added PSS is not consumed with a meal. Another mechanistic study investigated the gastrointestinal processes related to gastric emptying and gallbladder contraction [13]. A yoghurt drink consumed within 45 min before a main meal emptied faster from the stomach and did not sufficiently trigger gallbladder contraction as compared to consumption together with or 45 min after a main meal. Meal intake is thus important for triggering gallbladder contractility and stimulating bile flow as depicted in Figure 2. 

Another important aspect to consider is the amount of fat in the meal as it is generally believed that a certain threshold amount probably being in the range of 6 to 10 g fat is required to stimulate gallbladder contraction and subsequent bile flow [49]. Therefore, it can be assumed that a light breakfast like a serving of cereal with low-fat milk or yoghurt or a glass of milk and a cookie, may not contain enough fat to optimally trigger bile release, while a typical lunch or dinner meal or a heavy breakfast contains more fat (and protein), ultimately leading to a larger cholesterol-lowering effect than with (light) breakfast. Evidence from head-to-head studies testing this concept by comparing PSS intakes with different breakfast meals differing in their energy and fat content, would provide further insights into the impact of meal intake and composition on their cholesterol-lowering efficacy.

## 5. Impact of Age

The landmark meta-analyses of Law et al. [50] and Katan et al. [3] reported larger reductions of absolute LDL-C concentrations with PSS intakes in older individuals suggesting that efficacy would increase with age; LDL-C was for instance lowered by 0.21, 0.28, 0.31, 0.39 and 0.41 mmol/L in individuals aged 4–6, 20–29, 30–39, 40–49, and 50–60 years, respectively [3]. However, the relative reduction in LDL-C did not differ across these age groups. Hence, absolute reductions in LDL-C are larger in older individuals, but relative reductions remain comparable across age groups when taking their baseline concentrations into account. This outcome has been further supported by more recent meta-analyses [5,51] that clearly report no significant impact of age while describing that the ‘age effect’ is explained by higher baseline LDL-C concentrations which are known to increase with increasing age [52,53]. Therefore, the LDL-C lowering efficacy of PSS is not influenced by age and individuals with different ages will benefit from PSS intake with a comparable relative reduction in LDL-C concentrations.

## 6. Impact of Gender

As stated by others, there seems to be no difference by gender in the cholesterol-lowering effect of PSS [54]. This conclusion is based on several clinical studies that describe no difference in the LDL-C lowering effect of PSS between men and women [23,55,56,57,58,59]. However, most clinical studies investigating the cholesterol-lowering efficacy of PSS typically do not present serum lipid results separately for men and women, nor were studies designed and powered to assess a gender-specific difference. 

Other studies do suggest a possible influence of gender on the cholesterol-lowering effects of PSS. For instance, a clinical study that stratified participants by gender reported a different reduction in LDL-C between men and women [60]. A significant LDL-C reduction of 9.8% (*p* < 0.011) was found in men (*n* = 24) as compared to a non-significant 2.7% (*p* = 0.11) reduction in women (*n* = 38) after 3 weeks consuming 1.8 g/day of PSter [60]. In their meta-analysis based on individual data of PStan intervention studies, Naumann et al. [51] observed a trend for a difference in the LDL-C lowering effects of PStan in men compared to women suggesting that men were more responsive (*p* = 0.083 for an interaction of gender and PStan intake) [51]. It was further noted that this observation was not explained by differences in baseline LDL-C concentrations between men and women. Recently, two clinical studies again discussed a gender difference in the LDL-C lowering effect after PSS intervention [61,62]. In men consuming 2 g/day of PSter added to a breakfast serving of two wholegrain wheat cereal biscuits, total cholesterol and LDL-C concentrations were lowered by 9.5% and 9.8%, respectively, as compared to 2.1 and 3.6% in women [61]. Also, in a study using milk with 2.2 g/day of added PSter, apparently lower LDL-C concentrations in men than in women were found [62]. It should, however, be noted that these are small-scale studies with 45 or 54 individuals completing the study and are not powered to detect statistically significant differences between men and women. Hence, these suggested gender effects may just be chance findings. To further evaluate a possible gender impact, we have re-assessed the LDL-C lowering effects of PSter intervention based on previously reported large (*n* = 138–220) intervention studies [12,63,64] by reporting the outcomes for men and women separately (Table 4). Given a substantial impact of baseline LDL-C concentrations on absolute reductions, percent change in LDL-C are reported to take differences in baseline LDL-C concentrations between men and women into account. As summarized in Table 4, in none of these three studies a statistically significant gender by treatment interaction was found. Taking these findings together, there is no conclusive evidence of a gender-specific effect for the LDL-C lowering with PSS intake.

## 7. Summary and Conclusions

Numerous clinical studies have shown that the intake of PSS lowers LDL-C concentrations by 7.5 to 12% with daily intakes of 1.5 to 3 g. At intakes up to 3 g/day, PSter and PStan are equally efficacious. Data of PSS intakes above 4 g/day are scarce and do not allow a decisive conclusion on whether the dose-response relationship would continue. Further, it is also not clear whether the cholesterol-lowering efficacy would differ between PSter and PStan at higher doses. Because of the convincing evidence for their cholesterol-lowering benefit, PSS were amongst the first ingredients which received authorized health claims by regulatory bodies such as the European Food and Safety Authority (EFSA) and the US Food and Drug Administration (FDA). 

PSS are shown to be effective in various types of food formats such as fat-based foods like spreads, margarines as well as dairy-type foods and in food supplements including capsules and tablets, so facilitating choice for a sufficient intake of PSS to obtain their cholesterol-lowering benefit. There is no difference in efficacy between free and esterified PSS, nor does the choice of dietary fatty acid used for esterification or the source of the PSS have a meaningful impact. Compared to multiple daily intakes, a once-a-day intake of PSS, especially in the morning with a light breakfast, seems sub-optimal for lowering LDL-C, but seems influenced by intake occasion, i.e., with or without a meal and time of day. Meal intake and meal composition, i.e., fat content of a meal, are critical factors for an optimal LDL-C efficacy with PSS-added foods and food supplements. Consuming at least 2 g/day of PSS in the form of enriched foods or food supplements as an additional adjunct to a healthy diet are amongst the recommended dietary interventions for the management of dyslipidemia [65,66,67]. When consuming PSS, for an optimal cholesterol-lowering efficacy they should be consumed with a (main) meal such as a heavy breakfast, lunch or dinner and ideally twice daily.

## Figures and Tables

**Table 1:** Summary of the percent (%) LDL-cholesterol lowering effect of plant sterols and stanols (PSS) as described in meta-analyses.

Meta-Analysis	PSS Intake (Mean Dose or Dose Range) (g/day)	Number of Studies/Strata Included	Relative Reduction in LDL-C in % Plus 95% Confidence Interval (CI) in Brackets ()
Katan et al., 2003 [3]	0.7–1.1	8	−6.7 (−4.9; −8.6)
	1.5–1.9	13	−8.5 (−7.0; −10.1)
	2.0–2.4	14	−8.9 (−7.4; −10.5)
	≥2.5	21	−11.3 (−10.2; −12.3)
Demonty et al., 2009 [5]	2.15 *	141	−8.8 (−8.3; −9.4)
Musa-Veloso et al., 2011 [6]	2.63 (stanols) *	60	−10.3
	1.78 (sterols) *	120	−7.7
Ras et al., 2014 [7]	dose <1.0	24	−5.7 (−4.4; −7.1)
	≥1.0 dose <1.5	13	−6.4 (−4.6; −8.2)
	≥1.5 dose <2.0	55	−7.6 (−6.8; −8.4)
	≥2.0 dose <2.5	60	−8.4 (−7.6; −9.2)
	≥2.5 dose <3.0	17	−10.3 (−8.9; −13.6)
	≥3.0 dose <4.0	27	−12.4 (−11.2; −13.6)

* Refers to the mean daily intake based on all included studies/strata. PSS: plant sterols and stanols.

**Table 2:** Impact of intake frequency on the LDL-cholesterol (LDL-C) lowering effect of plant sterols and stanols (combined as PSS) as described in two meta-analyses.

Meta-Analysis	Intake Frequency	Average PSS Intake (g/day)	Number of Studies/Strata Included	Relative Reduction in LDL-C in % Plus 95% Confidence Interval (CI) in Brackets ()
Demonty et al., 2009 [5]	1/day	1.76 *	14	−6.1% (−4.1; −8.2)
	≥2 times/day	1.81 *	87	−8.9% (−8.1; −9.8)
Ras et al., 2014 ^#^ [7]	1/day	1.7	33	−6.9% (−5.7; −8.1)
	2-time/day	2.0	60	−8.4% (−7.5; −9.2)
	>2-time/day **	2.5	45	−10.0% (−8.9; −11.0)
				Statistically significant between intake groups (*p* = 0.001)

* Based on only studies/strata with an intake in the range of 1.6–2.0 g/day due to the small number of studies/strata with a single daily PSS intake outside of this dose range. ** Includes studies/strata with intakes of 2–3 and >2 times/day. **^#^** Based on post-hoc analyses of the data set from Ras et al., 2014 [7]. PSS: plant sterols and stanols.

**Table 3:** Impact of intake occasion (i.e., with a meal) plus intake frequency on the LDL-cholesterol (LDL-C) lowering effect of plant sterols and stanols (combined as PSS) based on meta-analysis data ^#^.

Intake Occasion	Average PSS Intake (g/day)	Number of Studies/Strata Included	Relative Reduction in LDL-C in % Plus 95% Confidence Interval (CI) in Brackets ()
Once a day at breakfast	1.7	9	−4.9% (−2.5; −7.2)
Once a day with another meal *	1.7	24	−7.6% (−6.2; −9.0)
More than once-a-day	2.2	105	−9.0% (−8.3; −9.7)
			Statistically significant between groups (*p* = 0.002)

* Refers to studies with once-a-day intake without specifying specifically breakfast as intake occasion ^#^ Based on post-hoc analyses of the data set from the meta-analysis of Ras et al., 2014 [7]. PSS; plant sterols and stanols.

**Table 4:** LDL-cholesterol (LDL-C) lowering effect of plant sterol (PSter) intake separated for men and women based on previously published intervention studies.

Study	PSter Intake (g/day)	Study Duration in Weeks	Study Population	Relative Reduction in LDL-C in % Plus 95% Confidence Interval (CI) in Brackets () as Compared to Control			Gender by Treatment Interaction
				Overall	Men	Women	
Trautwein et al., 2018 [12]	2	6	Individuals at risk of and with established T2DM	*n* = 138 −4.6 (−1.2; −8.0) *	*n* = 79 −5.8 (−1.4; −10.1) *	*n* = 59 −3.0 (+2.3; −8.1)	*p* = 0.414
Ras et al., 2015 [63]	3	4 8 12	Hyper-cholesterolemic healthy individuals	*n* = 220 −7.6 (−4.0; −11.0 * −8.2 (−4.9; −11.3) * −6.7 (−2.6; −10.5) *	*n* = 134 −6.8 (−2.2; −11.2) * −9.0 (−4.7; −13.0) * −6.9 (−1.7; −11.8) *	*n* = 86 −8.8 (−3.1; −14.3) * −6.9 (−1.5; −12.0) * −6.4 (+0.1; −12.5)	*p* = 0.582 *p* = 0.546 *p* = 0.901
Hendriks, et al., 2003 [64]	1.6	13 26 39 52	Healthy individuals	*n* = 185 −3.6 (+0.3; −7.3) −5.3 (−1.5; −8.9) * −6.0 (−2.3; −9.6) * −5.5 (−1.5; −9.4) *	*n* = 90 −4.3 (+1.2; −9.6) −5.7 (−0.4; −10.8) * −7.1 (−1.9; −12.1) * −6.7 (−1.0; −12.1) *	*n* = 95 −2.8 (+2.8; −8.1) −5.0 (+0.5; −10.1) −5.0 (+0.3; −10.1) −4.4 (+1.4; −9.9)	*p* = 0.694 *p* = 0.841 *p* = 0.575 *p* = 0.570

* Statistically significant compared to placebo, *p* < 0.05.
